# Slowing of fetal growth and elevated maternal serum sFLT1:PlGF are associated with early term spontaneous labor

**DOI:** 10.1016/j.ajog.2021.04.232

**Published:** 2021-11

**Authors:** Ulla Sovio, Francesca Gaccioli, Emma Cook, D. Stephen Charnock-Jones, Gordon C.S. Smith

**Affiliations:** a Department of Obstetrics and Gynaecology, University of Cambridge, Cambridge, United Kingdom; b NIHR Cambridge Biomedical Research Centre, Cambridge, United Kingdom; c Centre for Trophoblast Research, Department of Physiology, Development and Neuroscience, University of Cambridge, Cambridge, United Kingdom

**Keywords:** fetal growth, fetal stress, placental insufficiency, pregnancy, protein biomarkers, spontaneous labor

## Abstract

**Background:**

The physiological control of human parturition at term is unknown.

**Objective:**

This study aimed to test the hypothesis that slowing of fetal growth or elevated maternal serum levels of markers of placental hypoxia in late gestation will be associated with earlier term labor.

**Study Design:**

We observed 2208 women having first births and performed serial blinded ultrasonography and immunoassay of soluble fms-like tyrosine kinase-1 and placenta growth factor. We estimated the probability of spontaneous delivery from 37 weeks of gestational age concerning (1) fetal growth between 20 and 36 weeks of gestational age and (2) the maternal serum soluble fms-like tyrosine kinase-1–to–placenta growth factor ratio measured at approximately 36 weeks of gestational age. Data were analyzed using logistic regression and Cox regression.

**Results:**

Fetal size at 36 weeks of gestational age was not independently associated with the timing of delivery at term. However, there was an inverse relationship between fetal growth between 20 weeks of gestational age and 36 weeks of gestational age and the probability of spontaneous labor at 37 to 38 weeks’ gestation (hazard ratio [95% confidence interval] for a 50 percentile increase in abdominal circumference growth velocity, 0.60 [0.47–0.78]; *P*=.0001). This association was weaker at 39 to 40 weeks’ gestation (0.83 [0.74–0.93]; *P*=.0013), and there was no association at ≥41 weeks’ gestation. Very similar associations were observed for estimated fetal weight growth velocity. There was a positive relationship between soluble fms-like tyrosine kinase-1–to–placenta growth factor ratio and the probability of spontaneous labor at 37 to 38 weeks’ gestation (hazard ratio [95% confidence interval] for a 50 percentile increase in soluble fms-like tyrosine kinase-1–to–placenta growth factor ratio, 3.05 [2.32–4.02]; *P*<.0001). This association was weaker at 39 to 40 weeks’ gestation (1.46 [1.30–1.63]; *P*<.0001), and there was no association at ≥41 weeks’ gestation. Adjustment for maternal characteristics was without material effect on any of these associations.

**Conclusion:**

Slowing of fetal growth and biomarkers of placental insufficiency were associated with an increased probability of early onset of spontaneous term labor. We speculated that progressive placental insufficiency may be a physiological phenomenon that occurs with advancing gestational age near and at term and promotes the initiation of labor.

## Introduction

Despite decades of research, the mechanisms controlling the onset of human parturition are unclear,^1^ whereas the mechanisms are well understood in many animal species. In rodents and some ruminants, pregnancy is maintained by progesterone from the corpus luteum, and labor is initiated by luteolysis. In sheep, activation of the fetal hypothalamic-pituitary-adrenal (HPA) axis results in increased cortisol in fetal blood, which induces the expression of 17-α-hydroxylase in the placenta.^2^ The enzyme converts progesterone to estrogens, and the resultant fall in progesterone and rise in estradiol in the maternal serum result in myometrial activation, hence labor. In contrast, in humans and nonhuman primates, there is no striking change in the maternal circulating levels of progesterone or estrogens before labor. Current hypotheses of the mechanisms leading to human labor include a functional loss of the effects of progesterone signaling, despite normal circulating concentrations,^1^ and local production of estrogens from elevated levels of androgenic precursors arising from the fetal adrenal.^2^

We and others^3^^,^^4^ have shown that spontaneous preterm labor is preceded by slowing of fetal growth in some cases. Here, we examined 2208 healthy pregnant women at approximately 36 weeks of gestational age (wkGA) and related the timing of subsequent spontaneous term delivery to (1) fetal growth, assessed by comparing a blinded, research ultrasound scan at 36 wkGA with clinically indicated fetal biometry performed at 20 wkGA, and (2) maternal circulating placental proteins, which are thought to reflect placental hypoxia.^5^^,^^6^ We have hypothesized that both slowing of fetal growth and biochemical markers of placental dysfunction would be associated with earlier onset of labor at term.

**AJOG at a Glance**

**Why was this study conducted?**

This study aimed to determine whether markers of placental insufficiency in late gestation are associated with earlier spontaneous term labor in women having first births.

**Key findings**

Ultrasonic measurements indicating slowing of fetal growth and protein biomarkers of placental insufficiency were associated with a higher probability of earlier spontaneous labor at term.

**What does this add to what is known?**

The findings have suggested that placental insufficiency may be a trigger for labor at term.

## Materials and Methods

### Study design and cohort

The Pregnancy Outcome Prediction (POP) study was a prospective cohort study of unselected nulliparous women with a singleton pregnancy who attended the Rosie Hospital, Cambridge, United Kingdom, between January 2008 and July 2012. Ethical approval was obtained from the Cambridgeshire 2 Research Ethics Committee (reference number 07/H0308/163). The details of the study have previously been described.^7^ Briefly, all 4512 recruited participants gave written informed consent, and 4212 of them were followed up from recruitment at around 12 wkGA through to delivery. Gestational age (GA) was based on ultrasound, and most women were dated at <14 weeks’ gestation by crown-rump length measurement. Fetal biometry was obtained from the anomaly scan at 20 wkGA and 2 blinded research scans at 28 wkGA and 36 wkGA, and blood samples were collected at all 4 visits. Maternal serum was analyzed for soluble fms-like tyrosine kinase-1 (sFlt-1) and placenta growth factor (PlGF) as previously described.^8^^,^^9^ Outcome data were obtained by individual review of participants’ clinical paper records and by linkage to the hospital’s electronic records. This is the first analysis of the timing of term labor using the POP study cohort.

### Exclusions

From among the 4212 participants who completed the study, we excluded all preterm births (<37 wkGA) and women who had spontaneous rupture of the fetal membranes but did not go into spontaneous labor. In addition, we excluded participants who had one or more clinically indicated scans at or after 34 wkGA or had their 36 wkGA research scan result revealed. Finally, we excluded participants who did not have fetal biometry available at 20 wkGA or 36 wkGA or did not have sFlt-1 and PlGF measurements available at 36 weeks’ gestation.

### Definition of parameters

We studied biometric measurements, including abdominal circumference (AC), femur length (FL), and estimated fetal weight (EFW), based on AC and FL at 36 wkGA, defined as EFW (g) = 10ˆ(1.304+0.05281×AC+0.1938×FL−0.004×AC×FL), where AC and FL are expressed in centimeters.^10^ These were converted into *z* scores adjusted for the exact GA at measurement. Growth velocity (GV) was calculated for all these measurements (EFWGV, ACGV, FLGV) as the difference between the *z* scores at 36 wkGA and 20 wkGA, as previously described.^8^ In addition, we analyzed protein biomarkers at 36 wkGA including sFlt-1, PlGF, and the sFlt-1:PlGF ratio measured using the cobas e411 electrochemiluminescence immunoassay system (Roche Diagnostics Ltd, Basel, Switzerland), as previously described.^9^ Before the analysis, sFlt-1 and PlGF concentrations were transformed into multiples of the median adjusted for GA, maternal weight, and storage time at measurement. All biomarker measurements were log transformed and converted into *z* scores.

### Definition of outcomes

We performed the analyses using 2 different approaches, namely, logistic regression and Cox regression, to determine whether they produced consistent results. The definition of the outcome for logistic regression was spontaneous delivery at <40 wkGA vs any type of delivery at ≥40 wkGA as a binary outcome. The definition of the outcome for Cox regression was spontaneous delivery at any GA as a time-to-event outcome, censoring elective deliveries at birth. Censoring, in this case, indicates that the subject had not experienced spontaneous labor up to the given stage in pregnancy, hence contributing to the denominator up to that point but not beyond. Spontaneous delivery was defined as birth by any means that were not preceded by induction of labor and was not a prelabor cesarean delivery. Conversely, nonspontaneous delivery was defined as delivery by any mode that was not preceded by the spontaneous onset of labor.

### Statistical analysis

As a preliminary analysis, we explored the associations between the parameters and spontaneous delivery at <40 wkGA using fractional polynomials and Locally Weighted Scatterplot Smoothing (LOWESS) plots. Because of a small number of outliers in the *z* scores, some of the associations seemed U shaped. We mitigated the impact of overinfluential outliers by transforming the parameters into percentiles without imposing distributional assumptions (ie, 100 equal-sized groups). For descriptive purposes, we grouped the percentiles into deciles (10 equal-sized groups). Moreover, we explored the associations between the parameters and this outcome graphically by tabulating the odds on the log scale by decile and using the LOWESS approach on the percentiles instead of *z* scores.

We analyzed the associations between the parameters (as percentiles) and spontaneous delivery at <40 wkGA using logistic regression and reported the results as odds ratios (ORs).

In the analysis of spontaneous delivery at any GA, we chose GA as the time scale and applied censoring at birth to deliveries that were not preceded by the spontaneous onset of labor. We assessed the proportionality of hazards by examining log-log plots of survival, Nelson-Aalen cumulative hazard plots, and scaled Schoenfeld residual plots, and we performed a formal statistical test on Schoenfeld residuals. We drew smoothed hazard estimate plots to illustrate the instantaneous probability of spontaneous delivery throughout gestation at term in the 2 extreme deciles and deciles 2 to 9 of the parameter variables. The estimated smoothed hazard function curves included values near the extremes (37 wkGA and 42 wkGA), but the kernel estimates of the hazard function are generally less reliable in the boundary regions. Because of this and the high proportion of elective deliveries in the later GAs, we presented the smoothed hazard curves only up to 41 wkGA. We used Cox regression to estimate the associations between the parameters (as percentiles) and spontaneous delivery at any GA and reported the results as hazard ratios (HR) with 95% confidence intervals (CIs). In addition, we presented a Cox model where we have split the GA into 3 categories (37–38, 39–40, and ≥41 completed weeks) to address nonproportionality of hazards (see below). These models included an interaction between the GA category and the parameter, and the HR (95% CI) is presented for each GA category.

Maternal age, height, body mass index (BMI), education, deprivation index, previous miscarriage, ethnicity, marital status, smoking status, and fetal sex were considered as possible confounding variables in the analysis of both outcomes. Education, deprivation index, and ethnicity each contained a small proportion of missing values and were initially imputed with the mean or the most common value. To assess the extent of bias arising from the simple imputation method, we applied multiple imputation using chained equations (MICE)^11^ as an alternative method. We analyzed the associations between the potential confounders and the parameters using linear regression and the associations between the potential confounders and the outcomes using logistic regression and Cox regression. Of the 10 variables, only maternal age, BMI, and ethnicity were associated with both the model parameters and outcomes. We performed the final analyses with and without adjustment for these 3 characteristics, using the 2 methods of imputation for ethnicity (the only characteristic that included any missing values). We presented the estimates from the simpler method of imputation for ethnicity as it produced nearly identical results to MICE. Finally, we examined the interactions between the most strongly associated biometric measurements and biomarker measurements on the outcomes and assessed whether fetal biometry and biomarkers have independent associations on the outcomes.

## Results

**Figure 1:**
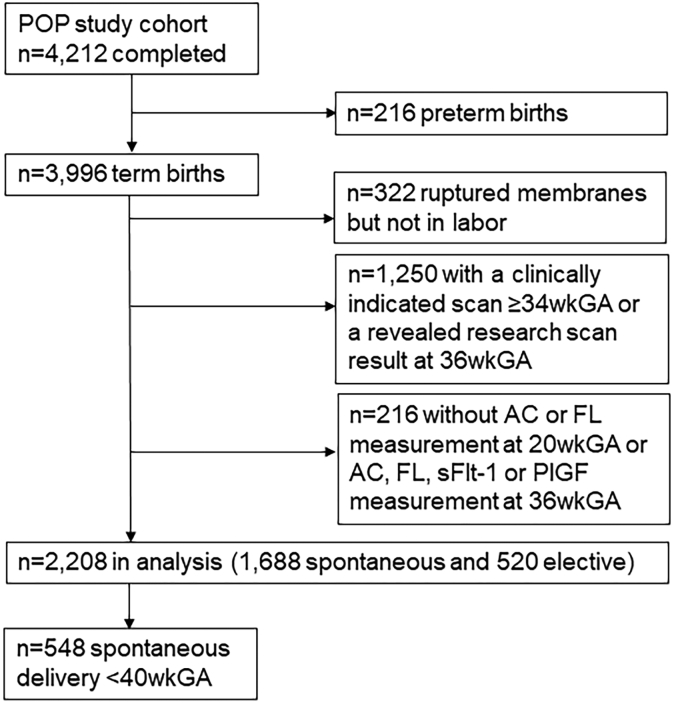
Flowchart of the selection of participants for the analysis *AC*, abdominal circumference; *FL*, femur length; *PlGF*, placental growth factor; *POP*, Pregnancy Outcome Prediction; *sFlt-1*, soluble fms-like tyrosine kinase-1; *wkGA*, weeks of gestational age. *Sovio et al. Slowing of fetal growth and elevated maternal serum sFLT1:PlGF ratio and early term labor. Am J Obstet Gynecol 2021*.

The selection of participants is described in Figure 1. Of the 4212 women who completed the study, 216 were excluded because of preterm birth. Furthermore, we excluded 322 women who had spontaneous rupture of membranes but did not go into spontaneous labor and 1250 women who had one or more clinically indicated scans at or after 34 wkGA or had their 36 wkGA research scan result revealed. Finally, of 4212 women, 216 (5%) were excluded because of missing data on AC, FL, sFlt-1, or PlGF. Moreover, 2208 women were included in the current analysis. Of these women, 1688 (76%) had a spontaneous delivery at any term GA, including 548 women who delivered spontaneously before 40 wkGA. The remaining 520 (24%) women had an elective delivery at any term GA. The characteristics of the study participants are described in Table 1.^12^ The women who had a spontaneous delivery before 40 wkGA were more often from a non-White ethnic background, and they delivered lighter babies than the rest of the women. The women who had an elective delivery had, on average, a higher BMI, and they delivered heavier babies at a later GA than the rest of the women.

**Table 1:** Characteristics of the population included in the analysis by the outcome

Characteristic	Spontaneous delivery at <40 wkGA (n=548)	Spontaneous delivery at any GA (n=1688)	Elective delivery at any GA (n=520)	All deliveries (N=2208)
Age (y)	30 (27–33)	30 (27–33)	30 (27–34)	30 (27–33)
Height (cm)	165 (161–170)	166 (161–170)	165 (160–168)	165 (161–170)
BMI (kg/m^2^)	23 (22–26)	23 (22–26)	25 (23–28)	24 (22–27)
Age stopped FTE (y)	21 (18–23)	21 (18–23)	21 (18–23)	21 (18–23)
Missing	18 (3.3)	42 (2.5)	16 (3.1)	58 (2.6)
Deprivation index	8.9 (5.7–13.9)	8.9 (5.7–13.9)	9.0 (5.1–14.0)	8.9 (5.6–13.9)
Missing	27 (4.9)	80 (4.7)	24 (4.6)	104 (4.7)
Previous miscarriages	44 (8.0)	148 (8.8)	46 (8.9)	194 (8.8)
Ethnicity				
Non-White	37 (6.8)	96 (5.7)	20 (3.9)	116 (5.3)
White	501 (91.4)	1566 (92.8)	489 (94.0)	2055 (93.1)
Missing	10 (1.8)	26 (1.5)	11 (2.1)	37 (1.7)
Married	384 (70.1)	1155 (68.4)	352 (67.8)	1507 (68.3)
Smoker	32 (5.8)	76 (4.5)	30 (5.8)	106 (4.8)
GA at dating scan (wk)	12.6 (12.0–13.1)	12.6 (12.1–13.1)	12.6 (12.1–13.1)	12.6 (12.1–13.1)
Interval length between 20 and 36 wk scans (d)	112 (109–113)	112 (109–113)	112 (109–113)	112 (109–113)
GA at birth, completed weeks				
37	46 (8.4)	46 (2.7)	7 (1.3)	53 (2.4)
38	142 (25.9)	142 (8.4)	41 (7.9)	183 (8.3)
39	360 (65.7)	360 (21.3)	51 (9.8)	411 (18.6)
40	0 (0)	630 (37.3)	68 (13.1)	698 (31.6)
41	0 (0)	482 (28.6)	228 (43.8)	710 (32.2)
42	0 (0)	28 (1.7)	125 (24.0)	153 (6.9)
Birthweight (g)	3265 (3025–3501)	3455 (3180–3743)	3613 (3270–3928)	3490 (3200–3790)
Fetal sex: female	262 (47.8)	835 (49.5)	262 (50.4)	1097 (49.7)

Data are expressed as median (25th–75th percentile) or number (percentage) as appropriate. For fields where there is no category labeled missing, data were 100% complete. Maternal age was defined at recruitment, maternal weight (used in the calculation of BMI) was measured at the time of the 12-week scan, and maternal height was measured at the time of the 20-week scan. All other characteristics were defined by self-report at the 20-week interview, from examination of the clinical case record, or linkage to the hospital’s electronic databases. Deprivation was quantified using the Index of Multiple Deprivation 2007, which is based on census data from the area of the mother’s postcode. Spontaneous delivery at <40 wkGA was defined as the occurrence of spontaneous birth between 37 weeks+0 days of GA and 39 weeks+6 days of GA, and spontaneous delivery at any GA was defined as the occurrence of spontaneous birth any time from 37 weeks+0 days. Elective delivery at any GA was defined as the occurrence of birth any time from 37 weeks+0 days in the presence of induction of labor or elective cesarean delivery.

*BMI*, body mass index; *FTE*, full-time education; *GA*, gestational age; *wkGA*, weeks of gestational age.

Adapted from Noble et al.^12^

*Sovio et al. Slowing of fetal growth and elevated maternal serum sFLT1:PlGF ratio and early term labor. Am J Obstet Gynecol 2021*.

**Figure 2:**
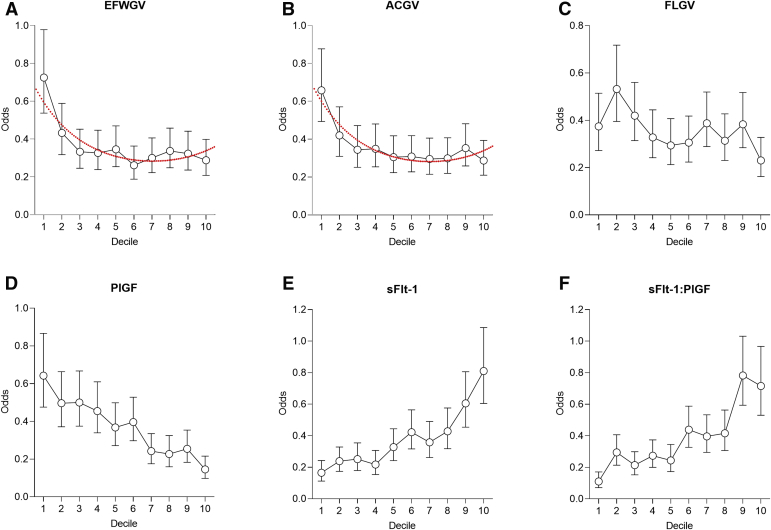
Spontaneous labor <40 weeks by deciles of growth and biomarkers Points are odds and bars are 95% confidence intervals. The dotted red lines in panels A and B represent the predicted odds from logistic regression models with polynomial (linear and quadratic) terms fitted on the percentiles of EFWGV and ACGV, respectively. The line on the figure is fitted to all of the observations and not on the values for the 10 deciles. *AC*, abdominal circumference; *EFW*, estimated fetal weight; *FL*, femur length; *GV*, growth velocity; *PlGF*, placental growth factor; *sFlt-1*, soluble fms-like tyrosine kinase-1; *wkGA,* weeks of gestational age. *Sovio et al. Slowing of fetal growth and elevated maternal serum sFLT1:PlGF ratio and early term labor. Am J Obstet Gynecol 2021*.

Univariable logistic regression analysis demonstrated that spontaneous delivery before 40 wkGA was associated with a smaller fetal size at 36 wkGA, slower GV between 20 wkGA and 36 wkGA and higher sFlt-1–to–PlGF ratio (higher sFlt-1 and lower PlGF) at 36 wkGA (Table 2). The inverse associations between EFWGV and ACGV and the log-odds ratio of spontaneous delivery at <40 wkGA were slightly nonlinear, suggesting a higher risk in the lowest decile of GV, whereas the direct association with sFlt-1–to–PlGF ratio was approximately linear, suggesting 2- to 3-fold higher odds per 50 percentile higher ratio (Figure 2, Table 2). Following adjustment for maternal characteristics, the associations with absolute fetal size were attenuated, and the estimates were consistent with no independent association. However, there were independent associations between spontaneous delivery at <40 wkGA and both fetal GV and the sFlt-1–to–PlGF ratio. When either EFWGV or ACGV was included in the logistic regression model simultaneously with the sFlt-1–to–PlGF ratio, independent associations of GV and the ratio were observed (Table 2).

**Table 2:** Associations with spontaneous delivery at <40 weeks of gestational age

Variable	Unadjusted OR (95% CI)	Adjusted OR (95% CI)
EFW at 36 wkGA	0.83 (0.69–0.98)	0.85 (0.72–1.02)
AC at 36 wkGA	0.83 (0.70–0.99)	0.86 (0.72–1.02)
FL at 36 wkGA	0.89 (0.75–1.06)	0.91 (0.76–1.07)
EFWGV 20–36 wkGA		
Linear term	0.73 (0.61–0.87)	0.75 (0.63–0.89)
Quadratic term	1.66 (1.19–2.31)	1.68 (1.21–2.34)
ACGV 20–36 wkGA		
Linear term	0.73 (0.62–0.87)	0.75 (0.63–0.88)
Quadratic term	1.69 (1.22–2.33)	1.69 (1.22–2.33)
FLGV 20–36 wkGA	0.79 (0.67–0.94)	0.80 (0.67–0.95)
PlGF at 36 wkGA	0.50 (0.42–0.59)	0.49 (0.41–0.59)
sFlt-1 at 36 wkGA	2.19 (1.83–2.62)	2.18 (1.82–2.61)
sFlt-1:PlGF ratio at 36 wkGA	2.34 (1.95–2.80)	2.36 (1.97–2.83)

ORs (95% CIs) from logistic regression analyses are presented per 50 percentile higher values in the parameter. Adjusted estimates are from the analysis adjusted for maternal age, body mass index, and ethnicity. For EFWGV and ACGV at 20 to 36 wkGA (centered at the mean), estimates from polynomial models, including linear and quadratic terms, are presented because of nonlinear associations. All analyses included 548 cases (spontaneous delivery at <40 wkGA) and 1561 noncases (any type of delivery at ≥40 wkGA). When either EFWGV or ACGV was included in the logistic regression model simultaneously with the sFlt-1–to–PlGF ratio (without adjustment for maternal characteristics), independent associations of GV and the ratio were observed. Estimated ORs (95% CIs) were 0.80 (0.67–0.95) and 1.65 (1.18–2.32) for the linear and quadratic terms of EFWGV, respectively, and 2.28 (1.90–2.73) for the sFlt-1–to–PlGF ratio. When ACGV was included instead of EFWGV, the associations were similar: 0.80 (0.68–0.95), 1.63 (1.17–2.27), and 2.27 (1.89–2.72), respectively.

*AC*, abdominal circumference; *CI*, confidence interval; *EFW*, estimated fetal weight; *FL*, femur length; *GV*, growth velocity; *OR*, odds ratio; *PlGF*, placental growth factor; *sFlt-1*, soluble fms-like tyrosine kinase-1; *wkGA*, weeks of gestational age.

*Sovio et al. Slowing of fetal growth and elevated maternal serum sFLT1:PlGF ratio and early term labor. Am J Obstet Gynecol 2021*.

**Figure 3:**
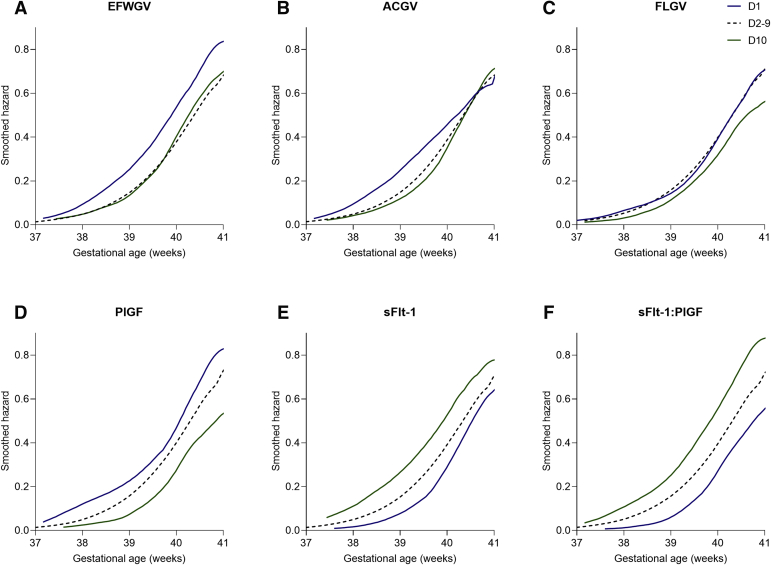
Smoothed estimated hazard function curves by deciles The blue lines represent decile 1, the black dashed lines represent deciles 2 to 9, and the green lines represent decile 10. *AC*, abdominal circumference; *EFW*, estimated fetal weight; *FL*, femur length; *GV*, growth velocity; *PlGF*, placental growth factor; *sFlt-1*, soluble fms-like tyrosine kinase-1. *Sovio et al. Slowing of fetal growth and elevated maternal serum sFLT1:PlGF ratio and early term labor. Am J Obstet Gynecol 2021*.

Univariable Cox regression analysis demonstrated that spontaneous delivery at any GA was associated with slower GV and higher sFlt-1–to–PlGF ratio (Table 3). However, the Cox model provided direct evidence that the magnitude of the associations differed across the range of GA, that is, some of the hazards were significantly nonproportional, and this was most evident for sFlt-1, PlGF, and the sFlt-1–to–PlGF ratio (Table 3). Adjustment for maternal characteristics demonstrated that there were independent associations between GV and maternal serum biomarkers but not absolute indicators of fetal size, consistent with the logistic regression analysis. However, multivariable analysis did not resolve the nonproportionality of hazards (Table 3). Smoothed hazard estimate plots described the instantaneous risk of spontaneous delivery at all term GAs by categories of fetal GV and maternal biomarkers (2 extreme deciles and deciles 2 to 9) (Figure 3). These plots illustrated that the lowest decile of EFWGV and ACGV were associated with an increased risk of spontaneous delivery at the earlier weeks of gestation at term. Analysis of biomarkers demonstrated an effect across the whole range of values, with higher risks of spontaneous delivery at the earlier weeks of gestation at term among women with lower PlGF, higher sFlt-1, and higher sFlt-1–to–PlGF ratio.

**Table 3:** Associations with spontaneous delivery at any gestational age

Variable	Unadjusted HR (95% CI)	HR *P* value	PH test *P* value	Adjusted HR (95% CI)	HR *P* value	Global PH test *P* value
EFW at 36 wkGA	0.89 (0.82–0.97)	.008	.41	0.92 (0.84–1.00)	.06	.44
AC at 36 wkGA	0.89 (0.81–0.97)	.006	.39	0.92 (0.84–1.00)	.05	.43
FL at 36 wkGA	0.96 (0.88–1.04)	.29	.46	0.96 (0.89–1.05)	.36	.46
EFWGV at 20–36 wkGA	0.83 (0.76–0.90)	<.0001	.02	0.84 (0.77–0.92)	.0001	.09
ACGV at 20–36 wkGA	0.85 (0.78–0.93)	.0002	.002	0.86 (0.79–0.94)	.0005	.01
FLGV at 20–36 wkGA	0.90 (0.83–0.98)	.01	.08	0.90 (0.83–0.98)	.02	.21
PlGF at 36 wkGA	0.73 (0.67–0.79)	<.0001	<.0001	0.72 (0.66–0.78)	<.0001	.0001
sFlt-1 at 36 wkGA	1.37 (1.26–1.49)	<.0001	<.0001	1.36 (1.25–1.48)	<.0001	<.0001
sFlt-1–to–PlGF at 36 wkGA	1.46 (1.34–1.59)	<.0001	<.0001	1.48 (1.36–1.61)	<.0001	<.0001

HRs (95% CIs) from Cox regression analyses are presented per 50 percentile higher values in the parameter. Adjusted estimates are from the analysis adjusted for maternal age, body mass index, and ethnicity. All analyses included 1688 cases (spontaneous delivery at any GA) and 520 noncases (elective delivery at any GA, censored at the time of delivery).

*AC*, abdominal circumference; *CI*, confidence interval; *EFW*, estimated fetal weight; *FL*, femur length; *HR*, hazards ratio; *GA*, gestational age; *GV*, growth velocity; *PH*, proportional hazards; *PlGF*, placental growth factor; *sFlt-1*, soluble fms-like tyrosine kinase-1; *wkGA*, weeks of gestational age.

*Sovio et al. Slowing of fetal growth and elevated maternal serum sFLT1:PlGF ratio and early term labor. Am J Obstet Gynecol 2021*.

Furthermore, we split the Cox regression analysis by GA into 3 categories (37–38, 39–40, and ≥41 completed weeks) for the parameters that showed evidence for nonproportionality of hazards: EFWGV, ACGV, sFlt-1, PlGF, and the sFlt-1–to–PlGF ratio (Table 3). The hazards were proportional when the interaction between the parameter and the GA category was included in the model. We found (Table 4) that slowing of fetal growth was most strongly associated with the risk of labor at 37 to 38 wkGA and that the association was attenuated at 39 to 40 wkGA and disappeared altogether at ≥41 wkGA. The pattern was broadly similar concerning placental biomarkers, with indicators of placental dysfunction (low PlGF, high sFlt-1, and sFlt-1–to–PlGF ratio) being associated with higher risks of labor at 37 to 38 wkGA, a smaller but still significantly increased risk at 39 to 40 wkGA, and a weak (PlGF) or no association (sFlt-1 and sFlt-1–to–PlGF ratio) at ≥41 wkGA. Adjustment for maternal characteristics did not essentially change the results (Table 4).

**Table 4:** Associations with spontaneous delivery at any gestational age, split into 37 to 38, 39 to 40, and ≥41 weeks of gestational age

Variable	37–38 wkGA HR (95% CI)	39–40 wkGA HR (95% CI)	≥41 wkGA HR (95% CI)
Unadjusted analysis			
EFWGV at 20–36 wkGA	0.65 (0.50–0.84)	0.80 (0.72–0.90)	0.97 (0.83–1.14)
ACGV at 20–36 wkGA	0.60 (0.47–0.78)	0.83 (0.74–0.93)	1.02 (0.87–1.19)
PlGF at 36 wkGA	0.43 (0.33–0.56)	0.74 (0.66–0.82)	0.85 (0.73–1.00)
sFlt-1 at 36 wkGA	3.26 (2.47–4.30)	1.39 (1.24–1.55)	0.97 (0.83–1.14)
sFlt-1–to–PlGF at 36 wkGA	3.05 (2.32–4.02)	1.46 (1.30–1.63)	1.12 (0.96–1.32)
Adjusted analysis			
EFWGV at 20–36 wkGA	0.67 (0.52–0.87)	0.82 (0.74–0.92)	0.97 (0.83–1.14)
ACGV at 20–36 wkGA	0.61 (0.48–0.79)	0.84 (0.76–0.94)	1.01 (0.86–1.19)
PlGF at 36 wkGA	0.42 (0.32–0.55)	0.73 (0.65–0.81)	0.84 (0.72–0.98)
sFlt-1 at 36 wkGA	3.28 (2.48–4.33)	1.39 (1.24–1.55)	0.95 (0.81–1.12)
sFlt-1–to–PlGF at 36 wkGA	3.11 (2.36–4.09)	1.48 (1.32–1.65)	1.13 (0.97–1.33)

Unadjusted and adjusted HRs (95% CIs) from Cox regression analyses are presented per 50 percentile higher values in the parameter showing some evidence for nonproportional hazards. Adjusted estimates are from the analysis adjusted for maternal age, body mass index, and ethnicity. HRs were proportional when GAs were divided into 37 to 38, 39 to 40, and ≥41 weeks (range of global proportionality of HR test *P* values:.57–.99). All analyses included 1688 cases (spontaneous delivery at any GA) and 520 noncases (elective delivery at any GA, censored at the time of delivery). When mutually adjusted for each other (but not for maternal characteristics), the HR (95% CI) for EFWGV was 0.74 (0.57–0.96) at 37 to 38 wkGA, 0.83 (0.74–0.93) at 39 to 40 wkGA, and 0.98 (0.84–1.16) at or after 41 wkGA, and for the sFlt-1–to–PlGF ratio, it was 2.93 (2.22–3.86), 1.43 (1.28–1.60), and 1.12 (0.95–1.31), respectively.

*AC*, abdominal circumference; *CI*, confidence interval; *EFW*, estimated fetal weight; *FL*, femur length; *GA*, gestational age; *GV*, growth velocity; *HR*, hazard ratio; *PlGF*, placental growth factor; *sFlt-1*, soluble fms-like tyrosine kinase-1; *wkGA,* weeks of gestational age.

*Sovio et al. Slowing of fetal growth and elevated maternal serum sFLT1:PlGF ratio and early term labor. Am J Obstet Gynecol 2021*.

## Discussion

### Principal findings

The main finding of this analysis was that slowing of fetal growth and an elevated sFlt-1–to–PlGF ratio at 36 wkGA were associated with an increased probability of spontaneous labor up to 40 wkGA. The relationship with slowing of fetal growth was apparent by comparing the relative size of the fetus at 36 wkGA with the relative size at 20 wkGA. Although the absolute size of the fetus at 36 wkGA was not independently associated with the subsequent timing of labor, fetuses that demonstrated a reduction in relative fetal size (expressed as change in z score) throughout the 2 assessments were more likely to deliver early. Furthermore, when we looked at the range of slowing in growth, there was a dose-dependent association: the greater the slowing in growth, the stronger the association. This was observed both when we compared a single biometric measurement (ie, AC) and when we analyzed growth using an EFW. In addition, we found that elevated sFlt-1 and low PlGF were associated with earlier delivery. Importantly, we addressed the hypothesis using 2 distinct analytical methods, and we observed very similar patterns of results.

In trophoblast-like cells, sFlt-1 messenger RNA is raised in response to hypoxia, and this is mediated by a hypoxia-inducible factor 2α–dependent mechanism.^5^ The levels of the transcripts encoding sFLT-1 and PlGF are both sensitive to placental stress.^6^ The sFlt-1 produced by the placenta is released into the maternal circulation and binds and inactivates the PlGF and other proangiogenic growth factors.^13^ In addition, it acts as a dominant negative antagonist of endothelial vascular endothelial growth factor receptors and sensitizes them to inflammatory cytokines.^14^ An elevated sFlt-1–to–PlGF ratio is associated with overtly pathologic situations, such as preeclampsia^15^ and fetal growth restriction,^16^ and we have previously reported that these complications were associated with placental histopathologic abnormalities in the POP study cohort.^17^ Therefore, we concluded that the current evidence supports the hypothesis that placental insufficiency near term may result in the promotion of early-term labor.

### Results

We and others have previously shown associations between early-onset slowing of fetal growth and the risk of spontaneous preterm birth.^3^^,^^4^ An interesting feature of this study is that we demonstrated these associations at term, when labor is physiological. Despite this, however, it is recognized that even delivery at 37 wkGA and 38 wkGA is not completely normal. Infants born at the earlier weeks of term are more likely to experience both short- and long-term complications.^18^^,^^19^ Hence, it is possible that the current associations are a logical extension of our previous findings with early-onset slowing of fetal growth and preterm birth. However, we also observed associations between these measurements and the probability of spontaneous labor up to 40 weeks’ gestation, which is clearly a physiological time for labor to occur. It is well recognized that the risk of many serious perinatal complications, such as stillbirth, increases at term and after term.^20^ Before the widespread use of induction of labor after term, there was the concept of the dysmature fetus and placenta after term.^21^ We have previously shown that fetal growth rate exceeds placental growth rate between 37 wkGA and 42 wkGA,^22^ and we speculate that advancing GA at term is associated with an increasing mismatch between fetal demands and placental supply. If this was correct, it could provide a potential mechanism for the current observations. Experimental studies in animals have indicated that interventions to produce relative placental insufficiency result in fetal hypoxia and hypoglycemia, which in turn cause activation of the fetal HPA axis,^23^ and there is evidence in human studies that fetal HPA activation may promote preterm labor.^24^ We speculated that physiological, relative placental insufficiency may result in activation of the fetal HPA axis, which in turn triggers labor. Clearly, there is potential for fetal compromise if the given degree of placental and fetal stress does not trigger labor. Furthermore, we speculated that the progressively increased risk of intrauterine fetal death beyond 39 weeks’ gestation^20^ might be explained, in part, by the failure of the fetal HPA axis either to respond to fetal stress or, if activated, to induce parturition.

### Clinical implications

We have previously argued that studying complications near term is justified by the clinical epidemiology of serious adverse outcomes of pregnancy.^25^ Most preeclampsias with severe features are associated with term birth,^25^ and one-third of all stillbirths occur at term.^26^ In addition, the study has clinical significance concerning defining normal fetal growth. In fact, we recently compared 2 methods for estimating fetal weight, a single-center study from the 1980s in Texas^27^ vs a modern, large-scale international collaboration, InterGrowth21st (IG21).^28^ Each study had derived an equation to calculate the ultrasonic EFW and had defined a normal range on the basis of their study population. We compared the ability of the 2 approaches to predict extremes of birthweight in the POP study. Surprisingly, the Texan (Hadlock) methods performed better than the IG21.^29^ Most of the women in the Texan study were close to birth (<2 days), whereas the IG21 included women within 2 weeks of birth. As the fetus could gain weight in the 2 weeks between the scan and delivery, the IG21 study corrected the EFW for the duration of the interval using the GA-related changes in EFW in the whole population. This approach implicitly assumed that the growth rate of fetuses would be the same regardless of how close to birth they were. The current analysis has provided direct evidence that this assumption is unlikely to be true and this may explain the better performance of the Hadlock method.

### Strengths and limitations

The strengths of this study were that the results of the ultrasound scans were not revealed to the mothers or their attending clinicians and the analysis of sFlt-1 and PlGF was made after the delivery and, therefore, could not influence clinical management. The study has a number of limitations. First, women continued to have induction of labor, which could be done for a maternal request or clinical indication. We addressed this using time-to-event analytical methods, treating nonspontaneous delivery as censoring.^30^ In addition, we excluded women who had a clinically indicated ultrasound scan at or near term, and this will have excluded women having induction of labor because of clinical concerns about abnormal fetal growth. A further limitation was that the study was confined to a single center and the population consists mostly of nondeprived women of White European ancestry, and it would be valuable to test for these associations in other populations.

### Conclusions

Both slowing of fetal growth and circulating markers of placental insufficiency immediately before term are associated with an increased risk of early-term labor. We have interpreted these findings as providing evidence that early-term labor can be a marker of placental insufficiency. In addition, we have speculated that activation of the human fetal HPA axis triggered by the consequences of relative placental insufficiency may have a role in the initiation of term labor.
